# Transactions of the Pathological Society of Philadelphia

**Published:** 1839-10-12

**Authors:** 


MEDICAL EXAMINER.

DEVOTED TO MEDICINE, SURGERY, AND THE COLLATERAL SCIENCES.

No. 41.] PHILADELPHIA, SATURDAY, OCTOBER 12, 1839. [Vol. II.

### TRANSACTIONS OF THE PATHOLOGICAL SOCIETY OF PHILADELPHIA.

*30th September*, 1839.

The President, Dr. Gerhard, in the chair.

Dr. Pennock exhibited to the Society a specimen of *Disease of the Heart, combined with Aneurism and extensive Ossification of the Aorta.* He also read details of the history of the case, and of the autopsy. Dr. Pennock requested and obtained permission to withdraw the paper, it being his intention shortly to publish it in connection with a series of cases on the subject.

*7th October*, 1839.

The Vice President, Dr. Pennock, in the chair.

Dr. Stillé exhibited a specimen of *Cancerous Tumour of the Foot*, and read the following notes of the case:

Mrs. Catharine C—— entered the Pennsylvania Hospital in the latter part of the month of September last. She was forty-seven years of age, the mother of twelve children, and her general health had always been robust. At least thirty years ago she was frequently struck with the tenderness of a particular part of the left instep; but she could not account for it, as there was no discoloration of the skin nor any swelling there. Fifteen years ago she let a heavy stick of wood fall upon the part, which was, in consequence, so badly bruised and swollen, that she was obliged to keep her bed for about a fortnight. As the effects of the accident disappeared she became sensible of a small lump, about the size of a pea, exactly over the spot which had formerly been tender to the touch, and which was now sensitive in an increased degree. This tumour became gradually larger until at last it occupied the whole back of the foot, extending from the ankle-joint to the toes, and from one side to the other of that member. The period of its most rapid increase has been the last two years, during which time it became larger in proportion over the *tarsus* than elsewhere. The pain in it has often been severe, and usually of a shooting or pricking character.

On Mrs. C——'s entrance into the hospital she was in the enjoyment of good general health; her appetite, digestion, and sleep, were natural; her strength was sufficient to enable her to perform without difficulty her household duties up to the period when she came to the city from the interior of Pennsylvania. Her menses began to become irregular about eighteen months ago, and ceased altogether within the last four months. The motions of the left foot upon the leg were free, but more limited than those of the right; the extension of the toes was more easily performed than their flexion. The tumour filled the space above described, and its highest point above the sole of the foot was about four inches. The skin covering it was of a dull red colour, and marked by many veins slightly distended. To the touch it was warmer than the neighbouring skin of the leg. The tumour, in general, was fixed and hard, though softer in its anterior than in its posterior portions; in the former of these it was elastic, and in points gave obscure signs of fluctuation: in these points the sensibility to pressure was acute. In several places could be felt resisting portions, as of bone.

Drs. Harris, Randolph, and Norris, having examined the case in consultation, it was agreed that the tumour was of the nature of osteo-sarcoma, and the patient was advised to have her leg amputated. She consented, and the operation was performed on the 4th October, by Dr. Norris. The patient is doing well at the present date.

*Examination of the tumour.*

The extreme limits of the tumour are as follow, viz., from the astragalus behind to the second phalanges of all the toes before; internally, from the internal malleolus to the first phalanx of the great toe; and externally, from the external malleolus to the second phalanx of the little toe. The arched form of the sole of the foot is preserved in a great degree, and the fat under the skin of this part, and that covering the heel, are abundant, and of a natural aspect. The tumour is enclosed in a proper fibrous sac, entirely distinct from the periosteum, which is perfect, except in certain points to be presently mentioned. This fibrous *envelope* is so distributed as to give distinct coverings to the several masses composing the tumour, and to their convolutions. The general aspect of the tumour, when divided by a longitudinal incision, bears a remarkable likeness to that of the brain, portions of the cut surface being of a pearly white, and others of different shades of gray, rosy, and red.

Several substances compose the tumour.

1*st.* An opaque, white, hard, elastic substance, generally disposed in rounded masses of irregular surface, grating under the scalpel, presenting, on section, a striated appearance, intermingled with a reddish gray substance, softer and less opaque than itself, and containing in the central portions of some parts shapeless masses of calcareous matter, of granular structure, and chalky whiteness. This substance forms the posterior third of the tumour.

2*d.* A cerebriform substance, which makes up the two anterior thirds of the tumour; it is of a grayish or rosy hue, is easily torn by firm pressure with the finger where most resistant, and is no where firmer than recent healthy brain; it varies from this degree of consistence to that of a soft pulp, easily removed by the handle of the scalpel. It is generally disposed in convoluted masses like the first.

3*d*. Numerous masses of osseous matter, superficial, or imbedded in the substance of the tumour, looking not unlike the *sequela* of necrosed bone, irregular in form, and from three to six lines in thickness. They have no connection with the skeleton of the foot.

4*th*. In several cavities situated in the anterior half of the tumour there are found, besides the softened medullary substance, coagula in the several stages of formation, viz. dark and clotted blood, amber-coloured, translucent masses, and opaque, brownish concretions, offering the fibrous structure well marked.

Of the bones of the foot, the metatarsal of the great, fourth, and little toes, have their ordinary shape, colour, and hardness; so also have the astragalus and the os calcis, and the phalanges of the toes. The articulations of these latter are in a healthy condition, and their motions are free. All the extensor tendons of the toes, and the flexors of the foot, pass through the body of the tumour, and move freely in it. The cuneiform, cuboid, and scaphoid bones, are not altered in shape, but are all more easily penetrated by the scalpel than in their sound state. The second and third metatarsal bones offer very profound alterations. The medullary canals in both are exposed, and contain a pulpy matter identical and continuous with that of the tumour. The second metatarsal bone is, in its upper third, a mere shell of semi-transparent bone, not thicker than a sheet of writing paper,—its sides swell out, so as to give the cavity, which is open above, an elliptical shape, with a transverse diameter of about half an inch. The lower two-thirds of this, which as well as the whole of the third metatarsal bone, are not more than two or three lines in thickness in the middle, present in points a spongy structure, are very brittle, of a whiter colour than the adjacent bones, and have their medullary canals exposed in several places. The periosteum appears to be wanting on both of these bones. The inter-osseous muscles are thin, pale, and flabby.

*Recapitulation.*

A woman of good constitution, without hereditary cancerous predisposition, has a tumour developed by a bruise in a spot habitually tender upon pressure. It increases during fifteen years; and the pain, together with the physical annoyance caused by the size of the tumour situated on her foot, induce her to have her leg amputated. After this operation, the tumour is found to consist of scirrhous and cancerous matters, and to have its most intimate connection with the medullary canals of two metatarsal bones.

*Observations.*

Benjamin Bell places the origin of osteo-sarcoma either in the bone, or in the adjacent soft parts, and makes its essential characters the conversion of these parts into a matter resembling flesh.* Dr. Warren, of Boston, says that, contrary to the opinion of most writers, he thinks osteo-sarcoma arises from the periosteum. He gives no description of the intimate structure of the tumours he treats of under this name. I have not been able to arrange this case under any of the descriptions given by English writers prior to the cultivation of pathological anatomy in France. It, however, answers perfectly to the disease described by Cruveilhier, La Roche and Sanson, and Begin, as scirrhus and carcinoma of the bones. Amongst the English authors, Drs. Carswell and Hodgkin have described the affection very accurately; the latter, especially, after treating separately of "scirrhus" and "fungoid disease," remarks that the two diseases are sometimes encountered in different organs of the same subject, as for example, there may be a scirrhus of the mamma, and a fungoid disease of the liver, spleen, or kidneys. "It sometimes, though less frequently, happens, that one part of a tumour has the character of true scirrhus, whilst another part is decidedly fungoid." Such, it is evident, is the combination existing in the present case.

That the tumour arose in this instance from within the second and third metatarsal bones seems probable, because, 1*st*, the tumour evidently began at the point where we now find the most serious lesion; 2*d*, the adjacent bones were comparatively but little injured; this would not have been the case if the more profound lesion alluded to had been merely the effect of pressure from without; 3*d*, these bones are changed in their entire structure; 4*th*, the matter contained in them is identical with that of the softer portions of the tumour.

The shell-like condition of the upper third of the second metatarsal bone resembles that of *spina ventosa;* but the characters of the tumour itself are strictly those of cancer, as it occurs in the glandular and other soft tissues, while the contents of a spina ventosa are mentioned as fluid. The existence of several pieces of bone in different parts of the tumour ought not, I think, to countenance the supposition of its being spina ventosa; for their mass (taken together) was greater than the deficiency in the metatarsal bones, to say nothing of their irregular shape, and their several positions.

In conclusion, then, it would seem that whatever name the tumour, as a whole, may receive, its anatomical characters were those of scirrhus and cancer. In this view, it is, indeed, not easy to account for the inconsiderable influence of the tumour on the health of the patient during the long period of fifteen years. But this is not a subject for discussion here.

---

M. Trousseau has been elected Professor of Materia Medica and Therapeutics in the Faculty of Medicine of Paris, in the place of the late Baron Alibert.

---

* A Treatise on the Diseases of the Bones, by Benjamin Bell. Edinb. 1828.

